# Nurse-led pathways for loneliness and social isolation in older adults: a narrative review of assessment, relational mechanisms, and implementation

**DOI:** 10.3389/fpubh.2026.1924348

**Published:** 2026-09-22

**Authors:** Xiaoxin Sun, Esteve Adrian Z. Estiva

**Affiliations:** School of Nursing, Philippine Women's University, Manila, Philippines

**Keywords:** implementation science, loneliness, nursing, older adults, public health, relational care, social isolation, social prescribing

## Abstract

Loneliness and social isolation in later life are major public health concerns that can compromise emotional wellbeing, participation, function, quality of life, and healthy aging. Intervention effects nevertheless remain heterogeneous and difficult to translate into routine services. This narrative review addresses a specific gap left by modality-based syntheses: how nursing assessment, relational work, and service implementation can be assembled into an accountable pathway from detection to sustained connection. A PubMed/MEDLINE search through 19 June 2026 was supplemented by focused publisher-record and citation-chain verification. Evidence was classified as nurse-led, nurse-delivered or supervised, transferable mechanism evidence, or implementation evidence. Direct nursing-role evidence remains limited by small samples, multicomponent interventions, inconsistent allocation and blinding, restricted populations, and short follow-up. Quantitative syntheses show average benefit but substantial between-study heterogeneity and possible small-study effects. We therefore propose a six-stage, review-derived pathway—detect and invite; differentiate and formulate; define a valued outcome; match and prepare; connect and verify; and review and adapt—with a separate failure-repair trigger rather than a seventh stage. The nursing contribution is described as clinical-relational integration: linking clinical assessment to the practical and interpersonal work required for an older adult to enter, test, and sustain a meaningful relationship or role. The pathway is a clinical heuristic, not a validated decision rule. Future studies should use hybrid effectiveness-implementation designs, report first-contact completion using an explicit denominator, assess relational harms, and examine equity and durability across settings.

## 1 Introduction

Loneliness and social isolation have moved from the margins of gerontology into clinical and policy discussions, but the terminology remains unstable in practice. Loneliness is a distressing discrepancy between desired and experienced relationships. Social isolation denotes an objectively restricted network, infrequent contact, or limited participation. Living alone is a household condition. These constructs overlap, yet none can be substituted for another: some people living alone are socially active and content, whereas others feel intensely lonely within a family household or care facility (1–3). This distinction is not semantic. It determines who is identified, which mechanism is targeted, and what counts as benefit.

The public-health relevance is substantial. Prevalence estimates vary with country, sampling frame, and instrument, and severe loneliness is less common than intermittent or moderate loneliness; nonetheless, both loneliness and objective isolation affect sizeable groups of older adults (4, 5). Longitudinal studies and meta-analyses associate these exposures with functional decline, cognitive deterioration, cardiovascular disease, health-care use, and mortality (6–12). These findings justify clinical attention, but they do not show that a short social program will reverse chronic disease. Residual confounding, bidirectionality, and differences in measurement remain important, particularly when distal biomedical outcomes are used to justify brief interventions (13, 14).

Within a healthy-aging perspective, social connection is not an isolated psychosocial endpoint. It interacts with emotional wellbeing, participation, functional capacity, activities of daily living, and quality of life. Recent observational evidence indicates that loneliness often clusters with stress, depressive symptoms, cognitive impairment, daytime sleepiness, pain, and residential-care exposure (15). A related educational commentary argues that health-professional curricula still give insufficient attention to recognizing and responding to loneliness (16).

The intervention literature has expanded without resolving this translational problem. Reviews pool programs as different as cognitive restructuring, group rehabilitation, exercise, volunteering, digital-skills training, befriending, home visiting, and community referral. Average effects are usually small to moderate, with marked heterogeneity in eligibility criteria, comparators, intervention content, and outcome measures (17–20). Participants are sometimes enrolled because they are older or live alone rather than because they report loneliness or restricted networks. In other studies, an intervention changes contact frequency but is evaluated only with a loneliness scale, or produces immediate score improvement without evidence that relationships persist. A modality-level question—“Do telephone calls work?”—therefore provides limited guidance unless the target, mechanism, dose, and implementation conditions are specified.

Recent reviews have improved estimates of average intervention effects, but they rarely resolve three questions that are central to nursing practice: who owns formulation and adaptation, which relational mechanism is expected to change, and whether the service can complete and sustain the first connection. Their conclusions are also constrained by heterogeneous eligibility criteria, inconsistent measurement of loneliness vs. isolation, sparse implementation data, and limited direct evidence in which nurses hold decision authority. Pooled modality effects therefore cannot substitute for a pathway-level account of target, mechanism, delivery responsibility, and failure repair.

Nurses are frequently proposed as part of the response because they encounter older adults in primary care, home care, chronic disease management, rehabilitation, hospital discharge, and long-term care. These encounters reveal bereavement, sensory loss, fear of falling, caregiver disruption, depression, poverty, and deterioration in informal support. Proximity, however, is not equivalent to efficacy. The direct nurse-led evidence base is modest, and many multidisciplinary programs do not report who formulated the social problem, supported the first contact, or responded when a referral failed. Claims that nursing is intrinsically superior would exceed the available evidence.

The knowledge gap is therefore not another catalog of telephone, group, digital, or social-prescribing interventions. It is the absence of a clinically accountable model linking differentiation of the target to mechanism selection, supported connection, reassessment, and organizational conditions. This review advances a circumscribed argument: nursing is most likely to add value through clinical-relational integration, combining assessment of health and functional complexity with the practical and interpersonal work that enables an older person to enter, test, and sustain a meaningful connection. Its original contribution is a three-layer evidence architecture and a six-stage pathway with an explicit failure-repair loop, first-contact completion as a mandatory process indicator, and a clear distinction between referral delivery and clinical benefit. The review addresses four questions: what should be assessed; which relational mechanisms plausibly influence outcomes; what direct and transferable evidence supports nursing practice; and what service conditions are required for implementation.

## 2 Review approach and evidence architecture

A targeted narrative search of PubMed/MEDLINE was undertaken from database inception to 19 June 2026. The principal query combined controlled vocabulary and title/abstract terms for older adults, loneliness or social isolation, and nursing or care-pathway concepts; the complete executable string, focused queries, dates, and restrictions are reported in Supplementary Data Sheet 1. English-language peer-reviewed reports were prioritized. Reference lists of major reviews and relevant trials were used for backward and forward citation chaining. Additional focused searches addressed emotional and social loneliness, the HEAL-HOA program, recent network meta-analysis, framework synthesis, realist-informed synthesis, and screening principles.

Supplementary Data Sheet 1 provides the executable PubMed query, source-verification flow, selection rules, author roles, evidence-layer definitions, framework-derivation procedure, and comparison with existing care frameworks. An independently documented CINAHL or PsycINFO search was unavailable. The direct nursing-role layer was therefore cross-checked against study lists from recent syntheses that searched CINAHL and/or PsycINFO, and potentially eligible reports were verified in PubMed or publisher records. This cross-database verification is not equivalent to an independently executed database search and remains a limitation.

The review was guided by the principles of the Scale for the Assessment of Narrative Review Articles (SANRA), particularly explicit aims, transparent source selection, design-sensitive interpretation, and presentation of data central to the argument (21). A narrative design was selected because the question required integration of construct measurement, heterogeneous intervention designs, qualitative implementation evidence, and theory-generating service architecture rather than estimation of a single pooled treatment effect. The traceable corpus comprised 59 publications. Focused searches and citation-chain verification identified 11 additional sources, yielding 70 publications in the final corpus. The initial search did not retain a machine-exported retrieval and screening log; consequently, the numbers retrieved, deduplicated, screened, and excluded cannot be reconstructed reliably and are reported as not recoverable. Supplementary Data Sheet 1 provides the executable PubMed query, restrictions, update boundaries, evidence-layer rules, and this reporting limitation.

Evidence was assigned using explicit role and inference rules. Direct nursing-role evidence comprised intervention studies in which a nurse, nursing student under nursing supervision, or nursing service had a documented role. Within this layer, studies were subclassified as nurse-led when nurses held assessment, coordination, or adaptation authority; nurse-delivered/supervised when nurses or nursing students delivered a protocol without clearly documented plan-adaptation authority; and nurse-involved when nursing participation was present but not the principal causal contrast. Transferable mechanism evidence comprised interventions delivered by lay workers, volunteers, other professionals, or mixed teams that tested mechanisms relevant to nursing practice without establishing a nurse-specific effect. Implementation evidence addressed workflow, acceptability, capacity, equity, fidelity, cost, or sustainability. Table 1 applies these rules study by study and adds a lightweight appraisal of allocation, blinding, attrition, outcome selection, and follow-up.

**Table 1:** Direct nursing-role evidence, role classification, and lightweight appraisal.

References	Nursing role and core function	Intervention and principal signal	Lightweight appraisal	Interpretive limit
Stewart et al. (51); demonstration project	Nurse-led: group delivery and relational continuity	Weekly public-health nursing groups up to 20 weeks; support satisfaction and positive affect improved; loneliness/isolation trends unclear	Nonrandomized; no blinded outcome assessment reported; small context-specific sample; multiple outcomes; short post-program assessment	No causal loneliness effect; broader psychosocial benefit should not be conflated with loneliness reduction
Theeke et al. (52); exploratory RCT; *n* = 27	Nurse-led: formulation and delivery of LISTEN	Meaning/cognitive-processing group intervention; lower loneliness and greater perceived support	Randomized with educational attention control; blinding not clearly feasible; approximately 13–14/arm; multiple outcomes without multiplicity adjustment; limited follow-up	Powered only for very large effects (about *d* = 1.12); no large independent replication
Ilgaz and Gozum (53); RCT; *n* = 69	Nurse-led: integrative assessment, delivery, and care planning	INOSEL group/person-centered program; large loneliness reduction and broader health signals	Randomized with routine-care comparator; participant/provider blinding infeasible; women-only, single setting; follow-up limited to intervention period	Large effect requires independent replication; component attribution impossible
Song and Boo (54); quasi-experimental; *n* = 126	Nurse-led: multicomponent coordination and delivery	Exercise, cognition, nutrition, and disease management; social support/activity improved	Nonrandomized comparison; no blinded delivery; not loneliness-specific; multicomponent outcomes; 12 weeks	Confounding and target mismatch limit loneliness inference
Noh et al. (55); prospective nonrandomized; *n* = 877	Nurse-involved integrated service: coordination role	Integrated health/social services; loneliness, frailty, and quality of life improved over 6 months	Nonrandom allocation; usual-service comparison; selection and loss to follow-up possible; broad outcome set	Cannot attribute change to nursing or a specific mechanism
Tseng et al. (56); single-blind RCT; *n* = 57	Nurse-led: hearing assessment and individualized management	Communication and hearing-aid satisfaction improved; loneliness effect not established	Randomized wait-list control; single-blind outcome assessment; low baseline loneliness; approximately 28–29/arm; 10 weeks	Inconclusive for loneliness; powered only for effects about *d* = 0.76
Hernandez-Lopez et al. (57); RCT; *n* = 119	Nurse-delivered/supervised: structured calls by nursing students	HELPeN calls; reported reductions in loneliness and social isolation	Randomized; delivery blinding infeasible; restricted context; limited durability and component analysis	Delivery evidence does not establish nurse-led adaptation authority
Hernandez-Ascanio et al. (58); cluster RCT; 63 intervention/56 control	Nurse-delivered/supervised: nursing students plus volunteers	Six home visits and five calls; no clear overall effect; selected emotional-loneliness/support signals	Cluster randomized; participant/provider blinding infeasible; per-protocol and intention-to-treat analyses; limited durability data	Comparable delivery authority to HELPeN; mixed workforce and selected signals limit inference
Thompson et al. (59, 60); qualitative implementation; same 8 nurses	Nurse-led implementation: assessment, co-production, referral, and follow-up	Feasible role; funding, workload, culture, family, and community capacity shaped delivery	Qualitative; no effectiveness allocation; same parent sample in two complementary reports; no clinical outcome follow-up	One contextual implementation source, not two independent effectiveness studies

A program was classified as nurse-led only when a nurse held primary responsibility for at least one core pathway function—assessment and formulation, intervention selection or adaptation, navigation and coordination, longitudinal review, or implementation leadership—and had sufficient authority to modify the care plan. Nurse-led does not mean nurse-only. Community organizations, volunteers, social-care staff, allied health professionals, family members, and mental-health clinicians may deliver essential components. The scope is adults generally aged 60 years or older in health or community-care settings; pharmacological treatment, population-wide campaigns, and interventions designed solely for younger adults were excluded.

Source selection and conceptual synthesis were conducted in two stages. XS screened titles, abstracts, and full reports for conceptual or evidentiary relevance and coded study design, delivery role, target construct, mechanism, pathway function, and implementation context. EAZE checked all records entering the direct nursing-role layer and all sources used to define the framework, challenged the proposed mappings, and resolved disagreements with XS by discussion. The conceptual outputs were derived through an iterative best-fit and realist-informed process: existing distinctions in loneliness measurement and complex-intervention theory provided the initial framework; recurrent context-mechanism-outcome patterns and pathway failures were then added inductively; discrepant studies were used to refine boundaries rather than discarded. This approach was informed by framework-synthesis and realist-reporting principles (22, 23). No external expert panel or older-adult stakeholder group validated the profiles or pathway, which remain review-derived hypotheses.

## 3 Defining the clinical target

A clinically useful formulation begins by separating subjective and objective dimensions. Loneliness concerns the perceived quality, intimacy, or availability of relationships. Objective isolation concerns network resources, contact, and participation. The two dimensions have distinct associations with health and may change independently (1, 24). A person may report less loneliness after one existing relationship becomes more dependable even if network size is unchanged. Conversely, an increase in participation may be a worthwhile outcome for an isolated person whose loneliness score remains stable. Studies that collapse both constructs into a single label of “social connection” risk obscuring these trajectories. These distinctions are also consistent with prior scoping work emphasizing that loneliness and social isolation overlap but are not interchangeable (25).

Living alone requires similar caution. It may identify exposure to practical vulnerability or a transition such as bereavement, but it is neither a diagnosis nor a reliable proxy for loneliness. Meta-analytic mortality associations for loneliness, isolation, and living alone differ in magnitude and are sensitive to study design and adjustment (11). Using household status as the sole eligibility criterion can recruit people who neither experience the target problem nor wish to alter their social life, while missing loneliness among people who live with others.

The intervention target is also more specific than “more contact.” Contact can be unwanted, conflictual, superficial, or exhausting. Older adults may lack opportunities, but they may instead lack an intimate confidant, a valued social role, confidence after repeated rejection, or accessible communication. A group program can increase exposure without creating belonging; psychotherapy can reduce threat appraisal without resolving transport; a hearing intervention can improve communication while leaving relationship opportunities unchanged. These examples explain why pooled modality effects are unstable and why assessment must identify the link in the causal chain that is most amenable to change (26, 27).

To support clinical reasoning, we derived six dominant disconnection profiles from the assessment, intervention, and implementation literature. An opportunity-deficit profile is characterized by few contacts, activities, or network resources. A relationship-quality profile involves available contacts that do not provide intimacy, recognition, or dependable support. A transition-related profile follows bereavement, retirement, relocation, hospitalization, caregiving change, or loss of function. An access-constrained profile reflects hearing or vision loss, mobility limitation, transport, cost, language, geography, venue design, or digital exclusion. A cognitive-affective profile includes depressive withdrawal, social anxiety, low self-efficacy, rejection sensitivity, or entrenched expectations of disconnection. A clinically complex profile combines loneliness or isolation with frailty, cognitive impairment, safeguarding concerns, severe mental illness, or unstable caregiving. The opportunity-deficit and relationship-quality profiles overlap with established social- and emotional-loneliness distinctions, respectively; transition-related disruption describes a temporal context, whereas access-constrained, cognitive-affective, and clinically complex profiles extend the taxonomy by identifying modifiable barriers and care complexity rather than claiming new loneliness constructs (28, 29).

These profiles are review-derived clinical heuristics, not validated diagnostic categories or biological phenotypes. They should not be used to assign treatment mechanically, and most people will span more than one. Their purpose is to make the treatment hypothesis explicit. An older adult who declines a day-center referral may be protecting against grief, unable to hear in groups, fearful of falling, unconvinced that the activity fits their identity, or recalling previous exclusion. Describing every refusal as low motivation converts a pathway failure into a patient deficit. A better formulation asks which barrier, expectation, or mismatch prevented the proposed connection from becoming usable.

## 4 Assessment: moving from detection to an actionable formulation

Case-finding has value only when a positive result can trigger a proportionate response. Universal screening is difficult to justify where services lack a pathway, community capacity, or clinical governance for severe distress. A more defensible starting point is targeted enquiry at high-yield moments: bereavement, hospital discharge, new functional or sensory loss, repeated non-attendance, transition to caregiving or living alone, worsening depression, or concern raised by the patient, family, or clinician (14, 30). The wording should normalize the topic and seek permission. Asking whether a person has as much companionship, closeness, or dependable support as they would like often elicits more useful information than a stigmatizing label. In programmatic terms, screening or routine case-finding is justified only when five conditions are met: an actionable target can be distinguished; a proportionate response pathway exists; the community sector has capacity to receive referrals; severe distress and safeguarding concerns are governed; and foreseeable burdens or harms can be monitored and managed (31). Targeted case-finding should be cross-referenced to the proportion approached, not only the proportion who completed assessment (Table 2).

**Table 2:** Six-stage pathway, failure-repair trigger, cross-cutting governance, and minimum monitoring requirements.

Pathway element	Core function	Primary denominator and timing	Minimum indicator/safeguard
Stage 1: detect and invite	Targeted enquiry at transitions or concern; seek permission	All eligible/approached; report at invitation	Proportion approached and accepting assessment; reason for decline; culturally acceptable language
Stage 2: differentiate and formulate	Paired loneliness/isolation assessment; emotional/social dimension; risk, barriers, preferences	All consenting/assessed; baseline	Documented formulation; urgent needs escalated; missing data and profile overlap
Stage 3: define a valued outcome	Record personally meaningful outcome and smallest feasible action	All formulated; baseline	Goal in participant language; agreed review horizon; no imposed social norm
Stage 4: match and prepare	Select mechanism-linked option; address symptoms, hearing, transport, cost, language and digital access	All with an agreed option; before connection	Barrier plan, readiness, accessibility, named contact, and community capacity confirmed
Stage 5: connect and verify	Warm handoff, supported first contact, feedback on welcome and fit	Primary: all for whom a referral/connection was initiated; 0–4 weeks	Referral-to-contact time; first-contact completion; experience; reason for failure. Also report all approached and consenting denominators
Stage 6: review and adapt	Review delivery, mechanism, and patient-reported outcomes; continue, step down, switch, escalate, or stop	All initiating a connection; 6–12 weeks/end of intervention; 6 and preferably 12 months	Repeat engagement; loneliness/network/goal change; switching reason; sustained participation; cost
Failure-repair trigger (not a seventh stage)	Clarify mismatch, barrier, deterioration, rejection, or unwanted contact; rematch/adapt/stop	All failed or unwanted connections; immediate review	Reason classified; participant preference reconfirmed; no automatic repetition; adverse experience documented
Cross-cutting governance and practice principles	Named pathway owner; boundaries; community capacity; supervision; safety; equity; data feedback	All pathway users and partners; continuous monitoring	Escalation for severe distress/safeguarding; continuity; community acceptance; nurse time; cost per sustained connection; relational harms; equity at each transition

Subjective loneliness can be measured with the 20-item UCLA Loneliness Scale or shorter versions. The three-item scale is feasible in busy settings and large surveys (32, 33). Objective network resources can be assessed with the six-item Lubben Social Network Scale, supplemented by questions about frequency and mode of contact, participation, availability of practical help, reciprocity, and conflict (34). Reviews of available instruments show that tools capture different constructs and are not interchangeable (35, 36). Neither the UCLA scales nor the LSNS-6 is a diagnostic test. Thresholds used for research vary, and a universal minimally important clinical difference has not been established across settings. Repeated measurement is most interpretable when the same version, administration mode, and language are retained. The De Jong Gierveld scale provides overall, emotional, and social loneliness scores and is particularly useful when the clinical question is whether intimacy/attachment or a broader network is missing (28). Direct single-item questions may be acceptable for initial enquiry but are sensitive to stigma and do not separate dimensions. The Social Provisions Scale can add information on attachment, social integration, reassurance of worth, reliable alliance, guidance, and nurturance, although it measures perceived provisions rather than loneliness itself (37). Instrument choice should therefore follow the formulation question, population, language, and mode of administration.

Paired assessment is preferable because a single score cannot identify the treatment target. A high loneliness score with an adequate network may indicate a relationship-quality or cognitive-affective problem. A restricted network with little distress may still matter if the person lacks emergency support, but intervention goals should not be imposed. Discordance between the subjective and objective measures is clinically informative rather than an error to be averaged away.

The score should be followed by a concise formulation. Five domains usually determine whether a connection pathway is actionable. First, establish onset and course: chronic, episodic, transition-linked, or recently worsening. Second, identify access constraints, including hearing, vision, pain, breathlessness, fatigue, continence, mobility, transport, affordability, language, caregiving duties, venue accessibility, and digital confidence. Hearing loss is particularly relevant because it is associated with loneliness and isolation, yet intervention must connect improved communication to an actual social opportunity (38). Third, examine the relationship context: bereavement, estrangement, conflict, perceived burden, lack of reciprocity, or loss of role. Fourth, assess mood, anxiety, avoidance, hopelessness, cognition, and social confidence. Fifth, clarify preferences: whom the person wishes to connect with, what role or activity would feel worthwhile, what intensity is acceptable, and what the smallest feasible step might be.

Clinical and safeguarding assessment remains separate from a loneliness scale. Severe depression, suicidal ideation, abuse or neglect, delirium, psychosis, substance-related harm, acute caregiver breakdown, and substantial cognitive impairment require established clinical or statutory pathways. A community activity cannot substitute for assessment and treatment. Cognitive impairment does not automatically exclude a person from social intervention; it may require supported decision-making, involvement of a trusted person, environmental adaptation, and different outcome measures.

Outcome selection should follow the causal hypothesis. We recommend three levels. Proximal outcomes establish whether the pathway operated: contact attempted, first contact completed, acceptability, safety, and barrier resolution. Intermediate outcomes test the intended social mechanism: repeat engagement, participation, network resources, perceived support, loneliness, or goal attainment. Distal outcomes include depression, wellbeing, function, health-care use, and mortality. Distal changes may matter, but they require longer follow-up and stronger causal caution. An unchanged loneliness score after a failed first contact is a delivery failure; the same score after sustained attendance may indicate a weak mechanism or poor fit. Without process data, these explanations are indistinguishable (39, 40) (Table 3). For operational use, proximal delivery outcomes should be assessed during the first 0–4 weeks; intermediate mechanism and patient-reported outcomes at 6–12 weeks and at the end of active intervention; and durability at 6 months, with 12 months preferred where feasible. First-contact completion should use all participants for whom a referral or connection was initiated as the primary denominator; services should additionally report all approached and all consenting participants so that loss at earlier transitions is visible. A completed referral is a process event, not a clinical outcome.

**Table 3:** Assessment domains for a clinically actionable formulation.

Domain	Suggested approach	What it informs	Key cautions
Subjective loneliness	Direct, non-stigmatizing enquiry; UCLA 3-item for brief case-finding; longer UCLA versions when precision is needed	Perceived insufficiency in companionship, intimacy, or support; change over time	Not a diagnosis; no universal cutoff or minimally important difference across settings (32, 33, 35)
Objective isolation and network resources	LSNS-6 plus contact frequency/mode, participation, practical help, reciprocity, and conflict	Network deficit, participation target, emergency support	Living alone is not equivalent to isolation; conflictual ties may inflate counts (1, 34)
Onset and transition	Ask when the problem began and about bereavement, retirement, relocation, hospitalization, caregiving change, and functional loss	Brief transition-focused care vs. a longer pathway	Do not pathologize expected grief; persistence, distress, and function matter (25)
Access constraints	Hearing, vision, mobility, pain, fatigue, continence, transport, cost, language, venue and digital accessibility	Prerequisites for participation and delivery mode	Barrier remediation may be necessary but insufficient without a relationship opportunity (38, 56)
Cognitive-affective processes	Mood, anxiety, avoidance, perceived burden, self-efficacy, rejection expectations, cognition	Need for activation, psychological treatment, graded exposure, or supported decision-making	Escalate severe mental illness or acute risk; do not substitute a social referral for treatment (43)
Preference, identity, and role	Preferred people, activities, culture, communication mode, desired reciprocity, smallest feasible next step	Person–intervention fit and acceptable intensity	Declining an option may indicate poor fit or structural constraint, not low motivation (46)
Outcome and review plan	Specify proximal, intermediate, and distal outcomes; set a review date	Whether the pathway was delivered, the mechanism changed, and the result mattered	Referral completion is not a clinical outcome (39, 40)
Loneliness dimension	De Jong Gierveld overall, emotional, and social loneliness scores; clarify whether intimacy/attachment or a broader network is missing	Maps emotional loneliness to relationship quality and social loneliness to opportunity/network deficit	Validated subscales do not replace formulation; cultural measurement properties may vary (28, 29)
Complementary perceived provisions	Single-item enquiry for invitation; Social Provisions Scale when attachment, integration, alliance, guidance, or nurturance is relevant (37)	Contextualizes why existing contacts do or do not meet relational needs	Direct items are stigma-sensitive; social provisions are not interchangeable with loneliness or objective isolation

LSNS-6, six-item Lubben Social Network Scale; UCLA, University of California, Los Angeles Loneliness Scale.

## 5 Relational mechanisms and the nursing contribution

The intervention literature often counts contacts as dose. That measure is convenient and incomplete. Contact becomes therapeutically relevant when it changes appraisal, agency, access, reciprocity, or relationship quality. Across modalities, five relational functions recur: continuity and attunement; supported agency; reciprocity and meaningful role; bridging; and remediation of barriers. These functions can be delivered by practitioners other than nurses. The nursing proposition is that a clinician who already understands symptoms, function, risk, and care transitions can integrate them into one pathway rather than treating the social problem as an external referral.

Continuity creates the conditions for accurate formulation. Loneliness is frequently accompanied by shame, perceived burdensomeness, or fear of rejection. A brief checklist may identify severity but not why previous attempts failed. Repeated contact with a named practitioner allows the account to become more specific and permits observation of small changes in mood, function, hearing, or support. Attunement is not generic empathy; it is the disciplined task of determining what form of connection is missing and what level of demand the person can tolerate. Continuity can itself reduce distress, but this effect has a boundary. A professional relationship that becomes the only dependable relationship may produce dependency or an abrupt loss when the program ends. The role should therefore be explicit, purposeful, and linked to wider sources of support.

Supported agency distinguishes an intervention from passive receipt of company. Earlier reviews observed that programs involving active participation and participant influence were more likely to report benefit than one-way provision of contact (41, 42). Mechanism-focused reviews likewise suggest that cognitive, behavioral, and social components matter more than the label attached to the program (27). Behavioral activation is a useful example: the practitioner helps the person identify a valued activity, anticipate obstacles, undertake a graded action, and review the result. In homebound older adults, tele-delivered behavioral activation produced more durable improvement in several social-connectedness indicators than friendly visits, although the intervention was delivered by lay counselors rather than nurses (43). The trial supports an active mechanism, not a nursing-specific effect.

Reciprocity and role address a different deficit. Programs framed entirely around receiving help may inadvertently reinforce passivity or stigma. Group rehabilitation, volunteering, peer support, creative production, mentoring, and mutual learning can restore the experience of contributing and being expected. In a randomized group rehabilitation trial, participants took part in goal-oriented activities and showed gains in social functioning and wellbeing, although results do not isolate reciprocity from group exposure and professional facilitation (44). The relevant process measure is not attendance alone but whether the person acquires a role, recognition, or a reason to return.

Bridging is the work between intention and participation. A conventional referral transfers information. A warm handoff transfers relationship and provisional responsibility. It may involve a named introduction, a three-way call, rehearsal of the first interaction, arranging transport, checking hearing or digital access, accompanying the person, and asking what happened afterwards. Social-prescribing reviews consistently describe assessment, individualized matching, and link-worker support as critical components, while overall effectiveness remains difficult to estimate because programs differ widely (45–47). For an older adult who anticipates rejection or cannot navigate eligibility rules, bridging may be the active ingredient.

Barrier remediation is necessary when disconnection is structurally produced. Hearing loss can make group conversation fatiguing; breathlessness or incontinence can make travel unacceptable; fees and transport can make an apparently available activity inaccessible. Digital tools may lower some barriers while creating others. The nursing role is to connect remediation to a social goal: hearing support followed by re-entry to a valued group, mobility rehabilitation followed by accompanied attendance, or digital coaching followed by a scheduled call with a chosen person. Treating the barrier without creating a relationship opportunity may improve a proximal clinical outcome while leaving loneliness unchanged.

Relational care also has ethical limits. Solitude may be chosen, and low contact is not inherently pathological. Cultural expectations concerning family, privacy, gender, and help-seeking affect whether a program is acceptable. Repeated outreach can feel intrusive, and failed group experiences can intensify exclusion. Consent, the right to decline, transparent boundaries, and the option to switch or stop are not peripheral implementation details; they are part of intervention fidelity (Figure 1).

**Figure 1:**
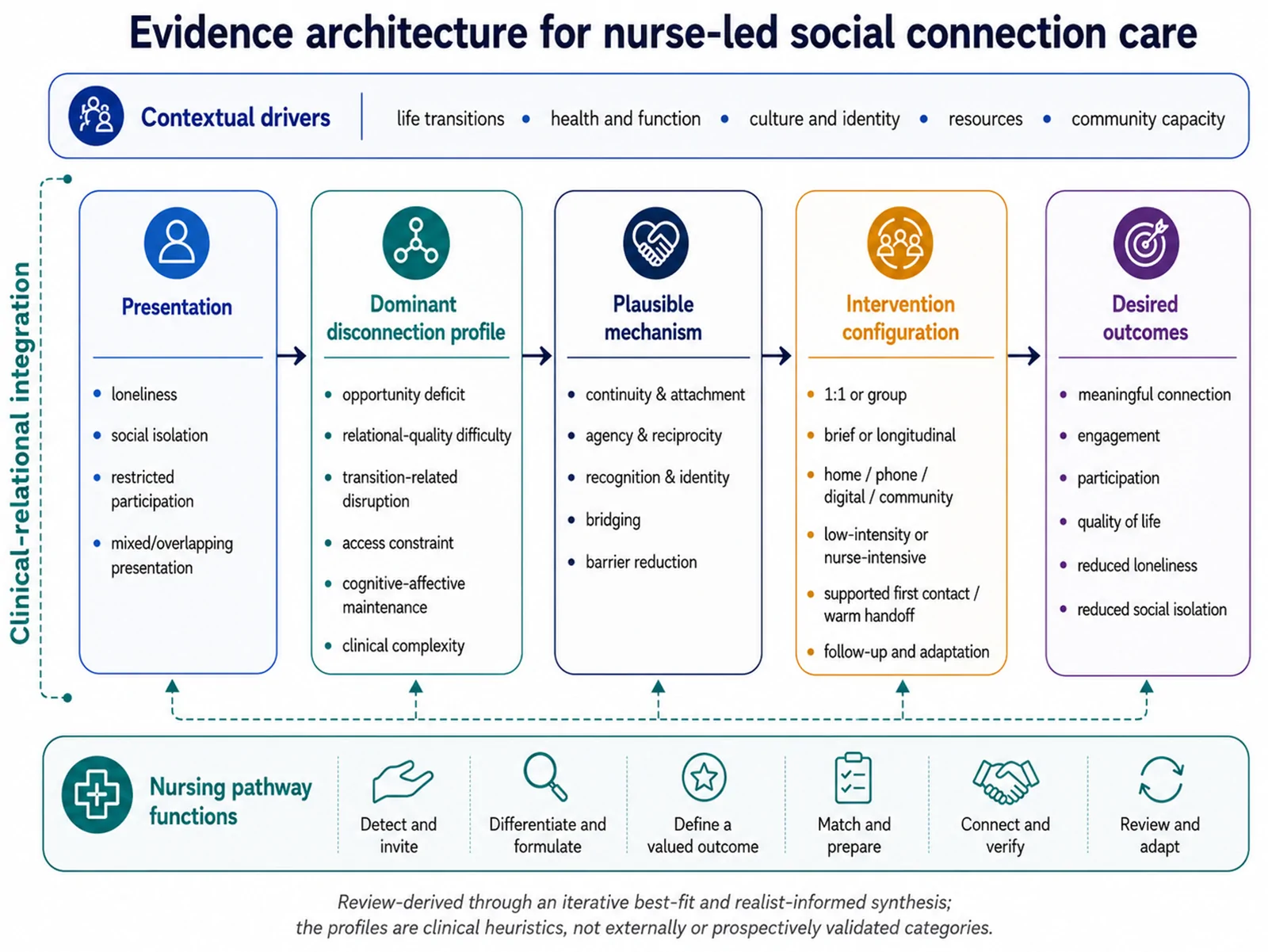
Evidence architecture for nurse-led social connection care. Contextual drivers shape a dominant disconnection profile, which should be linked to a plausible mechanism and intervention configuration. The nursing contribution spans the pathway through clinical-relational integration. The profiles are review-derived and require prospective validation.

## 6 What the intervention evidence supports—and what it does not

No review identifies a universally superior intervention. Meta-analyses report modest average reductions in loneliness for some group, psychological, and technology-supported approaches, but confidence is limited by small studies, heterogeneous comparators, variable baseline loneliness, and short follow-up (18–20). Home-based and community interventions can improve selected outcomes, although pooled estimates combine interventions with different mechanisms and populations (48, 49). The most defensible conclusion is conditional: an intervention is more likely to help when the enrolled population has the target problem, the mechanism matches the dominant barrier, and the pathway supports actual engagement.

Quantitative benchmarks sharpen this conditional interpretation (Table 4). Shekelle and colleagues estimated a small pooled effect for group-based randomized trials (SMD −0.25, 95% CI −0.42 to −0.08), while Duffner and colleagues reported an overall effect of *d* −0.47 (95% CI −0.62 to −0.32) with substantial unexplained heterogeneity. Coppola and colleagues reported SMD −1.11 (95% CI −2.19 to −0.03) after excluding high-risk studies, but *I*^2^ was 99.57%; a prediction interval derived from the reported variance is approximately −6.89 to 4.67 and crosses the null widely. Hoang and colleagues did not present one global pooled effect, and setting-specific estimates varied materially. Thus, a statistically significant pooled mean does not imply a stable effect in a new service.

**Table 4:** Quantitative benchmarks from key evidence syntheses.

References	Population/intervention grouping	Pooled effect (95% CI)	Heterogeneity/small-study signal	Prediction interval/interpretation
Hoang et al. (18)	No single global pool; setting/modality strata	Community technology SMD −0.19 (−0.51 to 0.14); long-term-care technology SMD −1.40 (−2.37 to −0.44)	*I*^2^ 59% and 70%, respectively; small categories limit funnel-plot power	No single numeric PI used here; materially different strata argue against one modality-wide effect
Shekelle et al. (19)	Group-based RCTs in community-living older adults	SMD −0.25 (−0.42 to −0.08)	Serious inconsistency; Egger test *p* = 0.01 for RCT pool, other bias tests nonsignificant	Numeric PI not reported in the main summary; average effect is small and not necessarily transportable
Duffner et al. (20)	27 intervention studies; *n* = 1,756	*d* −0.47 (−0.62 to −0.32)	Substantial unexplained heterogeneity; small-study effects assessed	Numeric PI not reported; unexplained heterogeneity limits service-level prediction
Coppola et al. (27)	25 RCTs after excluding high-risk studies	SMD −1.11 (−2.19 to −0.03)	*I*^2^ = 99.57%; Q (24) = 932.47; tau^2^ = 7.49; trend toward publication bias	Derived 95% PI approximately −6.89 to 4.67; crosses the null widely
Sun et al. (50)	Network meta-analysis; 46 quantitative studies, *n* = 6,049	Overall SMD −0.95 (−1.32 to −0.58)	Substantial clinical/design heterogeneity and indirect comparisons	Rankings are hypothesis-generating; they do not establish a universal intervention hierarchy

Small-study and reporting effects further temper interpretation. Shekelle et al. reported a significant Egger test for group-based randomized trials, although other bias tests were not significant. Coppola et al. found a trend toward publication bias in cognitive and social-behavioral activation interventions, with similar trim-and-fill estimates. Duffner et al. formally assessed small-study effects but substantial heterogeneity remained, and Hoang et al. used funnel plots within categories that often contained too few studies for reliable asymmetry testing. Recent network meta-analysis ranked psychosocial content and mixed delivery formats favorably, but a pooled SMD of −0.95 (95% CI −1.32 to −0.58) was drawn from heterogeneous direct and indirect comparisons; rankings should not be read as a universal treatment hierarchy (50).

### 6.1 Direct nurse-led and nurse-delivered evidence

The direct nursing literature is clinically informative but not yet definitive. An early public-health nursing support intervention for widowed older adults improved support satisfaction and positive affect, with trends rather than clear effects on social isolation or emotional loneliness (51). The finding is important because it separates broader psychosocial benefit from the loneliness endpoint; a supportive group can be valued without producing a large change on every social measure.

LISTEN (Loneliness Intervention using Story Theory to Enhance Nursing-sensitive outcomes) is one of the few interventions explicitly developed within nursing. In a randomized exploratory study of 27 chronically ill, lonely, community-dwelling older adults in Appalachia, the group receiving LISTEN reported lower loneliness and greater perceived social support at follow-up than an educational attention control (52). The intervention is conceptually stronger than nonspecific befriending because it addresses meaning, cognitive processing, and belonging. Its evidentiary weight is nevertheless limited by the very small sample, predominantly female participants, multiple outcomes, and lack of independent large-scale replication.

The INOSEL trial tested a group-based, person-centered program grounded in Integrative Nursing and the Omaha System among 69 older women with high loneliness. Loneliness declined in both groups, with a larger reduction in the intervention group, alongside improvements in physical activity, perceived health, social support, social inclusion, wellbeing, and spirituality (53). The large reported effect is hypothesis-generating rather than conclusive. A single setting, women-only recruitment, routine-care comparator, and multicomponent design make it difficult to determine how much benefit arose from nursing assessment, group exposure, walking, attention, or other components.

Broader nurse-led programs for older adults living alone provide indirect evidence for integrated pathways. A Korean quasi-experimental study of 126 prefrail or frail older adults living alone combined exercise, cognitive training, nutrition, and disease management; social support and activity improved over follow-up, but the intervention was not designed to isolate loneliness as the primary mechanism (54). A nonrandomized prospective integrated-service model involving 877 older adults living alone reported improvement in loneliness, frailty, and quality of life after 6 months (55). Both studies support the feasibility of nursing coordination across clinical and social needs, while nonrandom allocation, multicomponent content, and contextual specificity preclude a profession-specific causal claim.

The nurse-led Hear-Talk-Activity trial is better interpreted as inconclusive for loneliness rather than as a negative study. Fifty-seven older adults with hearing loss were randomized to a 10-week individualized program or wait-list control. Communication ability and hearing-aid satisfaction improved, whereas between-group differences in loneliness, depression, and quality of life were not demonstrated (56). Baseline loneliness was generally low, and with 28–29 participants per arm the trial had conventional 80% power only for an effect of approximately *d* = 0.76. The findings therefore show that the trial did not establish a loneliness effect; they do not exclude a moderate effect or prove that barrier remediation is insufficient.

The HELPeN program, delivered by trained nursing students under expert nursing supervision, evaluated structured telephone contact in 119 community-dwelling adults aged 65 years or older. The trial reported reductions in loneliness and social isolation and is notable for a specified training and supervision model (57). Replication in other systems, longer follow-up, and clearer component analysis are needed because telephone contact combines attention, continuity, monitoring, and possible activation. The result should not be generalized to unstructured calls. Using these role criteria, HELPeN is classified as nurse-delivered/supervised rather than clearly nurse-led because intervention delivery was specified but independent authority to reformulate or adapt the care plan was not documented.

Hernández-Ascanio and colleagues conducted a cluster-randomized trial in Spain with 63 intervention and 56 control participants. Six home visits interspersed with five telephone calls were delivered by nursing students and volunteers; the program did not demonstrate clear overall effects on isolation, loneliness, or health-related quality of life, although emotional loneliness and confidential support improved in selected analyses (58). Using the same role criteria, this study is classified as nurse-delivered/supervised. Its mixed workforce, cluster allocation, and per-protocol signals reinforce the need to separate delivery role from mechanism and causal certainty.

Implementation studies add an operational perspective that efficacy trials often omit. Australian general-practice nurses described assessment, goal setting, co-produced plans, referral, and follow-up as feasible elements of a social-connectedness intervention. Funding, workload, practice culture, adaptability, family support, and the strength of community infrastructure determined whether those elements could be sustained (59, 60). These qualitative studies do not demonstrate effectiveness; they identify the organizational conditions under which a nurse-led model may retain its intended relational functions (Table 1). References 59 and 60 report complementary analyses from the same parent sample of eight Australian general-practice nurses and are therefore treated as one implementation evidence source, not two independent studies.

### 6.2 Transferable mechanism evidence

Several trials delivered outside nursing clarify mechanisms that are directly relevant to nursing practice. The I-SOCIAL intervention combined individualized analysis of social barriers, counseling, group activities, and tailored suggestions for nearby activities. Loneliness declined relative to control after treatment and at 3 months (61). The study supports individualized formulation and social self-efficacy, but its multicomponent design prevents attribution to any single element.

Telephone contact varies sharply by content. During the COVID-19 pandemic, an empathy-focused program delivered by trained lay callers reduced loneliness, depression, and anxiety over a short period (62). Friendly attention may therefore have real value when it is structured, consistent, and responsive. By contrast, trials that compare psychologically active support with equally frequent friendly visits suggest that activation, goal testing, or cognitive work may be necessary for more durable change (43). Nursing services should specify what happens during a call rather than treating call frequency as the intervention.

The HEAL-HOA program provides a stronger, culturally distinct test of active telephone mechanisms. In 1,151 Hong Kong older adults who lived alone, were lonely, financially disadvantaged, and digitally excluded, eight 30-min sessions of layperson-delivered behavioral activation or mindfulness outperformed befriending on loneliness over short-term follow-up, with benefits sustained to 6 and 12 months in subsequent reports. At 12 months, social isolation partially mediated loneliness improvement (63–65). These findings support active mechanism and sustained follow-up, not a nurse-specific effect; they also show that telephone delivery can be relevant in a non-Western, resource-constrained subgroup when the intervention does more than provide receptive contact.

Taken together, these trials support a mechanism-based view. Continuity may be sufficient for transient distress or an acute transition; persistent loneliness associated with avoidance may require behavioral or cognitive work; objective isolation may require network opportunity and bridging; and loss of role may require reciprocal participation. None of these mechanisms is exclusive to nursing. Their relevance to nursing lies in the ability to select, coordinate, monitor, and revise them in the presence of clinical complexity.

### 6.3 Harms and adverse-experience reporting

A report-level audit of the eight effectiveness studies classified as direct nursing-role evidence found that none prospectively defined or systematically measured relational harms such as distress after rejection, burden from repeated outreach, dependency on the intervention relationship, or harm at abrupt termination. Safety information, when present, was generally limited to attrition or general clinical events. The absence of reported harm should therefore be interpreted as missing measurement rather than evidence of no harm. Future trials should prespecify adverse relational experiences, stopping rules, and procedures for unwanted contact.

### 6.4 Modality-level implications

Group programs offer repeated exposure, belonging, and reciprocal roles, yet they can fail when interest, language, functional capacity, or grief stage is mismatched. Meta-analytic averages conceal the fact that an inaccessible or culturally incongruent group delivers no dose. Preparation for the first session, a named welcome, observation of inclusion, and follow-up after non-attendance are therefore part of the intervention rather than administrative extras (44, 66).

Telephone and digital care can reach homebound or geographically isolated adults, but the medium has no intrinsic relational mechanism. Video-call evidence has been uncertain, while digital interventions show variable effects that depend on training, usability, and the presence of a meaningful person or community at the other end (67, 68). A digital pathway should assess device access, connectivity, hearing, vision, dexterity, cognition, privacy, and confidence, then define the social purpose. Hybrid options reduce the risk that service convenience is mistaken for patient access.

Social prescribing is best understood as a pathway design rather than a treatment label. Reviews consistently identify heterogeneity in eligibility, link-worker role, community assets, and outcome measurement (45–47). A directory or electronic referral may be useful for self-directed individuals with low barriers. For people with frailty, anxiety, low confidence, or complex eligibility, individualized matching and closed-loop support are more plausible mechanisms. Nurses cannot compensate indefinitely for underfunded community organizations; referral volume, waiting lists, transport, and accessibility must be visible to the clinical service.

One-to-one contact, befriending, and home visiting are often described as low-intensity interventions, but their intensity depends on content and duration. They may be appropriate for recent bereavement, homebound status, or as a bridge to wider participation. They are less likely to resolve chronic relationship-quality problems if the professional or volunteer becomes the only dependable contact. A planned transition, diversification of support, and clarity about endings are therefore essential.

Overall, the evidence does not support indiscriminate expansion of social activity. It supports better target selection, active engagement, individualized matching, and follow-through. Reviews that synthesize interventions by mechanism rather than by brand reinforce this interpretation, although certainty remains limited (27, 42, 69).

## 7 A review-derived nurse-led pathway

Figure 2 translates the synthesis into a six-stage pathway with a separate failure-repair trigger. It is intended as a clinical heuristic and service-design template, not a validated algorithm. Stage 1, detect and invite, uses routine contact or a transition to ask permission to explore loneliness or isolation. Stage 2, differentiate and formulate, pairs subjective and objective assessment, identifies a dominant disconnection profile, and separates a social need from urgent mental-health, cognitive, or safeguarding problems. Stage 3, define a valued outcome, records what the person wants to be different and specifies the smallest acceptable next step.

**Figure 2:**
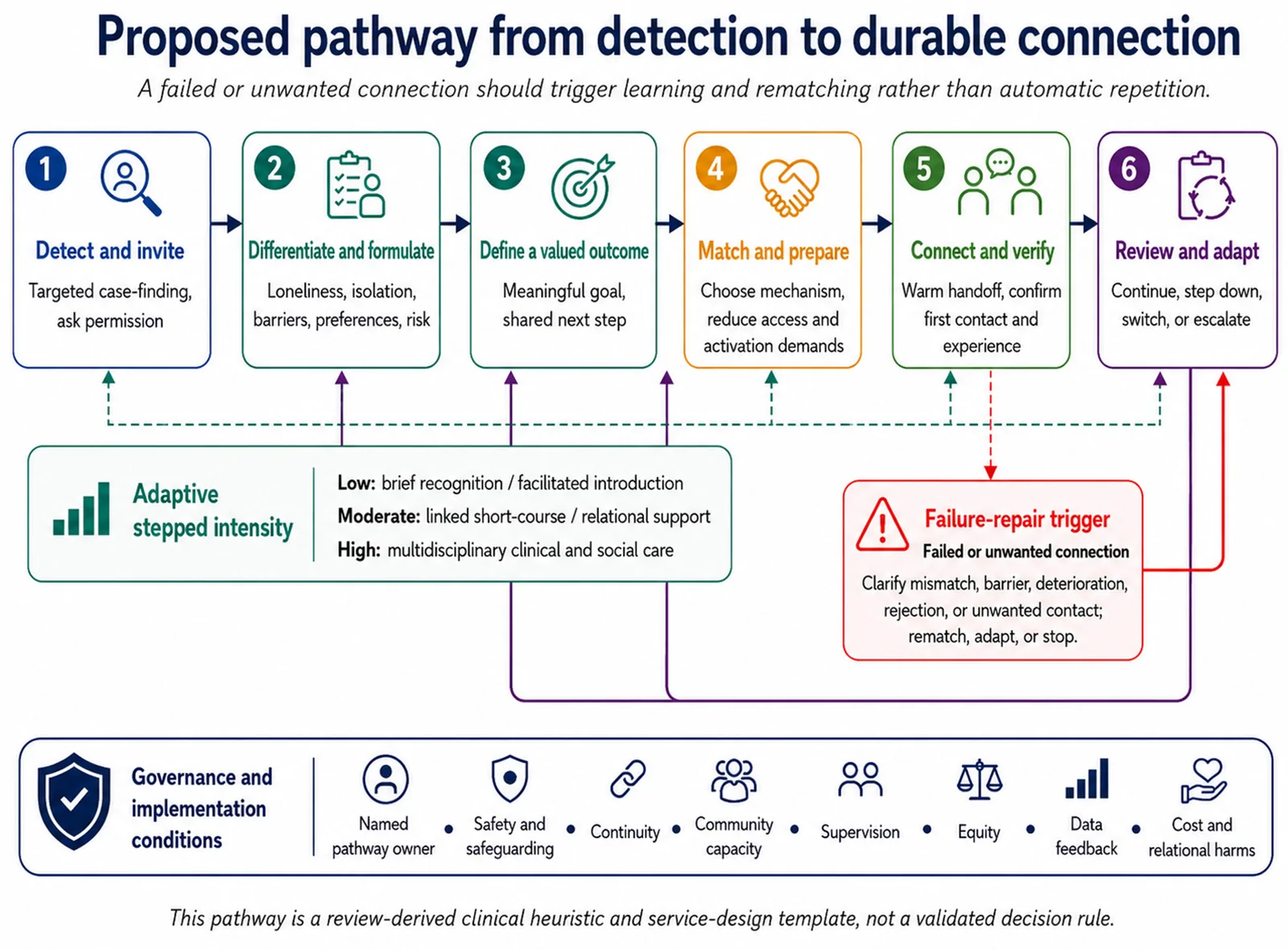
Proposed pathway from detection to durable connection. The figure shows six patient-facing stages. “Failed or unwanted connection” is a feedback trigger that routes the pathway to review, learning, and rematching rather than a seventh stage; stepped intensity and governance operate across stages. The pathway is a review-derived clinical heuristic, not a validated decision rule.

Stage 4, match and prepare, selects an option according to mechanism and preference. Preparation may include symptom stabilization, transport, hearing support, graded exposure, a script for the first call, or clarification of what the group expects. Stage 5, connect and verify, places a named person and a supported handoff between referral and participation. The nurse or connector confirms whether contact occurred, whether the person felt welcome, and whether another attempt is acceptable. Stage 6, review and adapt, reviews proximal delivery and patient-reported outcomes, then continues, steps down, switches, escalates, or stops. A failed or unwanted connection is not a seventh stage; it triggers clarification, learning, and rematching rather than automatic repetition.

A failed or unwanted connection is implementation data. It may reveal poor matching, an unaddressed barrier, inaccurate information, deterioration in health, or an intervention that the person never wanted. Repeating the same referral with more encouragement is rarely a rational response. The pathway should identify the failed link: invitation, access, first contact, inclusion, reciprocity, or maintenance. Repair may involve a different activity, a lower-demand first step, treatment of mood or hearing, another connector, or a decision to stop.

Intensity should be separated from modality. Low-intensity care may consist of brief formulation, reconnection with an existing relationship, or one facilitated introduction for a recent transition. Moderate-intensity care adds a named nurse or connector, repeated review, behavioral support, and active navigation. High-intensity care integrates mental-health, rehabilitation, geriatrics, social care, or safeguarding expertise for persistent loneliness with frailty, cognitive impairment, severe depression, unstable caregiving, or repeated pathway failure. Stepping is based on risk, complexity, access burden, and response—not a predetermined number of calls.

A minimally specified service requires more than “screen and refer.” It needs a paired assessment, a documented treatment hypothesis, a named pathway owner, current information on community eligibility and capacity, preparation for the first connection, a planned review, and a recovery route after non-engagement. These are functional requirements rather than a branded intervention package. Services unable to provide them should be cautious about routine screening because detection without response can document distress while offering no remedy (Figure 2; Table 2).

## 8 Implementation pathways across care settings

Implementation is part of the causal chain. Complex-intervention guidance emphasizes program theory, context, stakeholder perspectives, and iterative adaptation (40). In loneliness care, relevant context includes staffing continuity, appointment length, referral infrastructure, transport, information sharing, community-sector stability, and the acceptability of discussing social needs. A relationally sound program can fail when nurses have no protected time, community information is obsolete, or responsibility ends when an electronic referral is sent.

Primary care and community nursing offer longitudinal access to people with multimorbidity, but brief consultations and competing clinical priorities constrain formulation. Targeted case-finding, simple eligibility criteria, a named nurse or connector, and documentation that feeds back to the clinical record are more feasible than a universal screen detached from workflow (30). The Australian general-practice studies show that nurses can incorporate goal setting and social prescribing, but only when organizational culture, funding, role clarity, and community partnerships permit follow-up (59, 60). Minimum organizational requirements are protected assessment time, a named pathway owner, live referral information, documentation that returns outcomes to the clinical record, and authority to revise the plan after non-engagement. Without these, targeted enquiry should not be expanded into universal screening.

Home care permits observation of stairs, transport, hearing conditions, food insecurity, and the actual availability of support. It also increases the risk that the visiting professional becomes the principal social contact. Home-based pathways should distinguish therapeutic presence from bridging to wider relationships and should plan what happens when the service ends (48). Hospital admission and discharge are high-yield transitions because functional loss and disruption of routine may expose a fragile network. A transitional model—assessment before discharge, a named community contact, and review after return home—is more coherent than a one-time inpatient referral that cannot be monitored. Home-care services also require boundary planning and supervisory review because continuity can become dependency when the visiting professional is the only dependable contact. In hospitals, the pathway should specify who assumes ownership after discharge and a time-limited post-discharge verification point.

Residential and nursing care do not eliminate loneliness. Prevalence is substantial despite proximity to staff and other residents (70). Scheduled activities may increase occupancy without restoring autonomy or relationship quality. Interventions in these settings should address staff continuity, sensory and cognitive adaptation, family contact, resident choice, and opportunities to contribute. Residents with cognitive impairment should not be excluded solely because a standard self-report instrument is difficult to administer; observational, proxy, and goal-based outcomes may be needed. Long-term-care implementation depends less on adding activities than on stable staffing, resident choice, sensory accessibility, small-group fit, family participation, and the capacity to observe whether a resident is included rather than merely present.

Workforce development must match the proposed role. Nurses require skill in non-stigmatizing enquiry, interpretation of brief measures, formulation, shared decision-making, behavioral activation principles, cultural humility, sensory and digital accessibility, safeguarding, and local navigation. A single education session is unlikely to change practice without supervision, case discussion, protected time, and caseload limits. Repeated exposure to grief, deprivation, and failed referrals can create moral distress; supervision and clear boundaries are implementation requirements, not staff benefits added later.

Community capacity is a frequent hidden bottleneck. Referral pathways should include live information on eligibility, language, fees, transport, waiting times, accessibility, and whether the organization can accept further participants. Bidirectional communication is needed so that a declined referral or non-attendance returns to the pathway owner. Community partners should participate in service design and be resourced for the demand generated. Otherwise, a health service can improve its referral metric while transferring workload and risk to an unstable voluntary sector.

Equity should be examined at every transition in the pathway. People with low income, rural residence, minoritized language or culture, sensory impairment, cognitive impairment, unstable housing, or limited digital access may be least likely to be approached, accept, complete the first contact, and remain engaged. Reporting mean change among completers can make an inequitable pathway appear successful. Evaluations should stratify reach and benefit, document reasons for non-engagement, and provide interpreters, accessible materials, supported consent, transport, and hybrid delivery where necessary.

The evidence base remains dominated by high-income settings, and transfer to low- and middle-income contexts cannot be assumed. Multigenerational co-residence may coexist with emotional loneliness, while filial expectations, stigma, language, migration, gender roles, and informal-care norms can alter both disclosure and preferred support. Digital and community-sector assumptions are especially fragile where devices, connectivity, transport, or formal voluntary organizations are limited. The most culturally contingent stages are detect and invite—because acceptable language and who may ask differ—and match and prepare—because available community assets and family roles shape feasible options. Local co-design, culturally tested measures, and explicit reporting of community capacity are therefore prerequisites for adaptation, not optional refinements.

Fidelity should attach to functions rather than surface form. A home visit, telephone call, or video session may all preserve core functions if they provide valid formulation, co-produced goals, supported action, continuity, and feedback. Conversely, a manualized program can lose its mechanism when staff change at each contact or referrals are never reviewed. Implementation outcomes—acceptability, adoption, appropriateness, feasibility, fidelity, cost, penetration, and sustainability—should be reported alongside clinical outcomes (39). Determinants can be organized across intervention, inner setting, outer setting, individuals, and implementation process (71).

## 9 Discussion: controversies, evidence gaps, and research priorities

This review agrees with prior systematic reviews that intervention effects are heterogeneous and that no modality is reliably superior across populations. It differs in the unit of analysis. Hoang, Shekelle, Duffner, and Sun primarily compare intervention categories or delivery formats; Coppola organizes trials by hypothesized mechanisms; social-prescribing reviews examine referral models. This synthesis instead connects these findings to professional role, first-contact completion, failure repair, and service governance. The proposed pathway therefore does not claim a new therapeutic mechanism. Its added value lies in operational integration: distinguishing nurse-led from nurse-delivered work, making the failed connection a learning trigger, requiring an explicit denominator for first contact, and refusing to treat a generated referral as a patient outcome. Supplementary Table 3 compares these additions with social prescribing, the 5A structure, stepped care, and National Academies recommendations.

The first controversy is medicalization. Health-care involvement can identify treatable contributors and reach people who would otherwise be missed, but a clinical label can obscure poverty, ageism, inaccessible transport, loss of public space, and erosion of community infrastructure. The appropriate nursing response is neither to individualize every social problem nor to withdraw because causes are structural. Clinical-relational integration should connect individual assessment to community and policy constraints while making clear which problems cannot be solved by a referral.

The second controversy concerns outcomes. A statistically significant reduction in a loneliness scale may not represent a relationship the person values, while stable loneliness may coexist with restored participation or a meaningful role. Core outcome work should include subjective loneliness, objective connection, patient-defined goals, and sustained engagement. Adverse experiences also require reporting: distress after rejection, privacy concerns, burden from repeated contact, dependency on a professional relationship, and harm associated with abrupt termination. The field has focused more on whether scores improve than on whether an intervention can worsen the experience of exclusion. The direct-evidence audit in Section 6.3 shows that relational harms were not systematically measured, so claims of safety cannot be inferred from silence.

The third issue is attribution. Many programs are described as nurse-led because a nurse is present, yet the active components are not linked to professional competencies or time. Future studies should specify who completed formulation, how continuity was maintained, who could adapt the plan, how much time was spent on bridging, and what happened after failed contact. Comparisons may be more informative when they test an integrated pathway against information-only referral, or continuity against fragmented delivery, rather than nurse vs. non-nurse. Professional title should not be treated as a mechanism.

Durability remains underexamined. Relationships develop at variable speeds, and initial attendance does not establish belonging. Follow-up should extend beyond program completion and distinguish continued contact, continued participation, and continued benefit. Economic evaluation should include nurse time, supervision, transport, technology support, community-sector costs, and opportunity cost. Claims that social prescribing or volunteer contact saves health-care expenditure are premature without robust counterfactual data and longer follow-up (12). At minimum, studies should report 0–4-week delivery, 6–12-week mechanism and patient-reported outcomes, and 6-month durability; 12-month follow-up is preferred for sustained participation and relationship continuity.

The next generation of trials should be mechanism-informed and adaptive. Hybrid effectiveness–implementation designs can test outcomes while examining reach, fidelity, workload, and sustainability. Factorial designs could estimate the added value of warm handoff, continuity, behavioral activation, digital support, or booster contact. Sequential multiple-assignment designs could test switching rules when an initial intervention fails. Stratification should be prespecified for baseline loneliness vs. isolation, access constraints, living arrangement, cognition, transition context, and relevant cultural factors.

Measurement should follow the causal chain. A hearing intervention should demonstrate communication change and participation before a distal loneliness effect is expected. A network intervention should measure first contact and repeated interaction. A cognitive intervention should include appraisal, avoidance, or self-efficacy. Relational fidelity requires measures of continuity, perceived attunement, shared decision-making, reciprocity, and supported agency; otherwise, the proposed active ingredient remains invisible. Recent mechanism-focused synthesis is a useful advance, but primary studies still report intervention brands more consistently than mechanisms (27).

Recruitment must include those who are hardest to reach: rural residents, culturally and linguistically diverse groups, people with sensory or cognitive impairment, care-home residents, and adults with low income or limited digital access. Older adults and community organizations should participate in selecting outcomes and interpreting non-participation. Finally, trials need explicit stopping and switching rules. More of the same is not an evidence-based response to non-improvement.

## 10 Strengths and limitations of this review

This review contributes a design-sensitive synthesis that separates direct nursing evidence from transferable mechanisms and implementation evidence. It also reframes referral completion as an inadequate endpoint and makes the pathway between detection and sustained connection explicit. The disconnection profiles, endpoint hierarchy, and six-stage pathway with a failure-repair loop provide testable service-design propositions rather than a catalog of modalities.

Several limitations should temper interpretation. PubMed/MEDLINE and citation chaining formed the primary search base; the focused cross-database verification relied on study lists from reviews that had searched CINAHL and PsycINFO rather than independently executed searches, so nursing, psychological, social-care, occupational-therapy, and gray literature may still have been missed. The original narrative search did not retain a database-level screening log, preventing a retrospective PRISMA-style count without reconstruction. Source selection was not fully duplicated, and only the direct nursing-role layer received structured lightweight appraisal; no formal risk-of-bias tool or certainty grading was applied to the complete corpus. These choices increase susceptibility to selection and interpretation bias and mean that comparative confidence statements should be read as design-sensitive judgments, not GRADE ratings. The evidence is predominantly from high-income settings, intervention heterogeneity limits transportability, and 2025–2026 studies have little replication. The profiles and pathway were derived by two authors without external older-adult or expert validation and require prospective testing before use as decision rules.

## 11 Conclusion

Loneliness and social isolation in older adults are heterogeneous experiences produced by interacting relational, psychological, clinical, functional, and structural conditions. Screening alone does not resolve them, and no single modality is consistently superior. For aging and public health services, the evidence favors a shift from activity selection to pathway design: distinguish subjective loneliness from objective isolation, formulate the barrier and desired change, match a plausible mechanism, support the first connection, and learn from what happens next.

Nurses can contribute through clinical-relational integration, particularly when symptoms, frailty, sensory loss, risk, and care transitions make a social option difficult to use. Direct nursing-role studies are promising but remain too small, heterogeneous, and inconsistently explicit about decision authority to establish a superior professional model. The immediate research task is to test clearly specified, equity-sensitive pathways with functional and relational fidelity. Success is a connection that the older person can enter, values enough to repeat, and can sustain—not a completed screen, delivered call, or generated referral.
