# Supplementary material for: Nurse-led pathways for loneliness and social isolation in older adults: a narrative review of assessment, relational mechanisms, and implementation
Source: Front Public Health. 2026 Sep 22;14:1924348. doi: 10.3389/fpubh.2026.1924348 (PMC13639791; doi:10.3389/fpubh.2026.1924348)
Supplement: Supplementary file 1 [file Table_1.docx]

**Supplementary Material 1. Search, source selection, appraisal, framework derivation, and metadata audit**

# 1. Review purpose and design

The review was designed as a critical narrative synthesis rather than a systematic review or effect-estimation exercise. Its purpose was to integrate construct measurement, direct nursing-role evidence, transferable relational mechanisms, implementation determinants, and service-design implications. The heterogeneous evidence types and the theory-generating objective justified an iterative narrative, best-fit framework, and realist-informed approach.

# 2. Database, dates, and restrictions

The primary database was PubMed/MEDLINE, searched from database inception to 19 June 2026. English-language, peer-reviewed reports were prioritized. No restriction was applied by country or publication year. Pharmacological treatment, population-wide campaigns, studies designed solely for younger adults, and sources without direct conceptual, intervention, measurement, or implementation relevance were excluded. Reference lists of major reviews and eligible trials were used for citation chaining.

# 3. Reproducible core PubMed query

(("Aged"[Mesh] OR "Aged, 80 and over"[Mesh] OR older adult*[tiab] OR older people[tiab] OR ageing[tiab] OR aging[tiab]) AND ("Loneliness"[Mesh] OR loneliness[tiab] OR "social isolation"[tiab] OR "social disconnectedness"[tiab] OR "social connection"[tiab] OR "living alone"[tiab]) AND ("Nursing"[Mesh] OR nurs*[tiab] OR "Primary Health Care"[Mesh] OR "primary care"[tiab] OR "community care"[tiab] OR "home care"[tiab] OR "social prescribing"[tiab] OR telephone[tiab] OR digital[tiab] OR screen*[tiab] OR assess*[tiab] OR implement*[tiab]))

This string reconstructs the documented core logic in executable form. Because the original narrative search did not retain a machine-exported search history, it should not be interpreted as a retrospectively exact search-log artifact or as the basis of a PRISMA claim.

# 4. Focused update searches

- ("loneliness"[tiab] AND (nurs*[tiab] OR nursing student*[tiab]) AND (trial[tiab] OR intervention*[tiab]))
- ("emotional loneliness"[tiab] OR "social loneliness"[tiab] OR "De Jong Gierveld"[tiab]) AND (aged[tiab] OR older[tiab])
- ("HEAL-HOA"[tiab] OR (loneliness[tiab] AND behavioral activation[tiab] AND mindfulness[tiab]))
- (loneliness[tiab] AND older[tiab] AND "network meta-analysis"[pt])
- (loneliness[tiab] AND older[tiab] AND (harm*[tiab] OR adverse[tiab] OR unwanted[tiab]))
- (("framework synthesis"[tiab] OR realist synthesis[tiab]) AND (health care[tiab] OR nursing[tiab]))

# 5. Cross-database verification of the direct nursing-role layer

An independently documented CINAHL or PsycINFO search was not available for the original review and was not retrospectively claimed. To reduce the resulting risk, the direct nursing-role layer was cross-checked against eligible-study lists and bibliographies in recent systematic reviews that searched CINAHL and/or PsycINFO. Candidate nursing-role reports were then verified in PubMed or publisher records. This procedure improves recall but is not equivalent to independent database execution; the limitation is stated in the manuscript. The reviews used for this cross-check, the databases searched, and the last search date are reported in Table S1A. The most recent review used for the cross-check searched literature through 23 March 2023; reports published thereafter were addressed through the focused update searches in Section 4.

Table S1A. Reviews used for cross-database verification of the direct nursing-role layer.

| **Review used for cross-check** | **Databases searched** | **Last search date** |
| --- | --- | --- |
| Fakoya et al. 2020 (Ref. 26) | MEDLINE, Embase, and CINAHL; supplementary Google/Google Scholar and backward citation searching | 15 June 2018 |
| Hoang et al. 2022 (Ref. 18) | Ovid MEDLINE, CINAHL, CENTRAL, Embase, PsycINFO, Web of Science, and Scopus | March 2020 |
| Duffner et al. 2024 (Ref. 20) | MEDLINE, PsycINFO, and CINAHL via EBSCOhost | 23 March 2023 |

# 6. Numerical source-verification flow

Table S1B. Auditable corpus-update flow and reporting boundary.

| **Audit stage** | **Number** | **Reporting status / disposition** |
| --- | --- | --- |
| Original narrative corpus available for re-audit | 59 | All reports rechecked for role classification and current citation status |
| Additional sources retained through focused updating | 11 | Measurement (2), healthy ageing/education (2), HEAL-HOA (3), network meta-analysis (1), synthesis method (2), screening principles (1) |
| Final narrative corpus | 70 | 59 original sources plus 11 focused-update sources |

# 7. Source selection and author roles

XS screened titles, abstracts, and full reports for conceptual or evidentiary relevance and extracted study design, population, target construct, delivery role, mechanism, outcome, follow-up, and implementation context. EAZE checked all records assigned to the direct nursing-role layer and all framework-defining sources. Disagreements about inclusion, role classification, or framework mapping were resolved through discussion and revision of the relevant boundary rule. Selection was not fully duplicated for the complete narrative corpus.

# 8. Evidence-layer and nursing-role rules

Table S2. Evidence-layer and nursing-role classification rules.

| **Category** | **Operational rule** | **Permitted inference** |
| --- | --- | --- |
| Nurse-led | Nurse holds assessment, coordination, intervention-selection/adaptation, longitudinal-review, or implementation authority | Supports feasibility/effectiveness of a nursing-owned pathway function; does not prove nursing superiority |
| Nurse-delivered/supervised | Nurse or nursing student delivers a specified protocol, but independent authority to reformulate/adapt is not documented | Supports deliverability under nursing supervision; not a nurse-led adaptation effect |
| Nurse-involved | Nursing is one component of an integrated service without separable authority or effect | Supports multidisciplinary feasibility only |
| Transferable mechanism | Lay, volunteer, psychological, social-care, or mixed-team intervention tests a mechanism relevant to nursing | Supports mechanism plausibility, not a nurse-specific effect |
| Implementation evidence | Qualitative, process, feasibility, cost, or determinant study | Supports workflow/context interpretation, not clinical effectiveness |

# 9. Lightweight appraisal for direct nursing-role evidence

Each direct nursing-role study was appraised descriptively for design, allocation, feasibility of blinding, attrition or analysis population, outcome selection and multiplicity, follow-up duration, and the nursing function actually held. The appraisal was not converted into a summary score. Table 2 in the manuscript reports these domains and uses them to qualify confidence statements.

# 10. Derivation of the profiles, evidence architecture, and pathway

The synthesis combined deductive and inductive coding. Deductive starting categories were subjective versus objective disconnection, emotional versus social loneliness, complex-intervention program theory, and implementation outcomes. XS coded recurrent contexts, barriers, mechanisms, delivery functions, outcomes, and failure points. Recurrent patterns that did not fit the starting categories were added inductively. EAZE reviewed and challenged the mappings. Disconfirming examples were retained to refine boundaries. The six profiles, three-layer evidence architecture, and six-stage pathway were finalized by iterative consensus. The process was informed by best-fit framework synthesis and realist context-mechanism-outcome reasoning; it was not a full realist synthesis. No external expert or older-adult stakeholder validation was conducted.

# 11. Comparison with existing frameworks

Table S3. Comparison of the proposed pathway with existing frameworks.

| **Existing framework** | **Shared elements** | **What the present pathway adds** |
| --- | --- | --- |
| Social prescribing/link-worker model | Identification of social need, community referral, navigation | Clinical differentiation of loneliness/isolation; nursing-role boundary; mandatory first-contact verification; failure-repair loop |
| 5A structure | Ask/assess, assist, arrange, follow-up logic | Relational formulation, mechanism matching, community-capacity check, and explicit distinction between referral and outcome |
| Stepped care | Intensity matched to need and response | Stepping based on relational mechanism, access burden, risk, and failed connection rather than symptom score alone |
| National Academies recommendations | Recognition, assessment, referral, health-system responsibility | Operational denominators, relational harms, first-contact completion, and cross-setting governance |
| Current six-stage pathway | Detect; formulate; define valued outcome; match; connect; review | Failure is treated as learning data; six stages remain distinct from the failure trigger and cross-cutting governance |

# 12. Main limitations of the search and synthesis

- The primary database was PubMed/MEDLINE; cross-database verification did not replace independent CINAHL/PsycINFO searches.
- Grey literature and non-English reports may have been missed.
- The original narrative search did not retain a prospective database-level flow, and the update flow should not be read as PRISMA screening.
- Selection was not fully duplicated and no formal risk-of-bias or GRADE assessment was applied to the complete corpus.
- The evidence is dominated by high-income settings, and cultural transferability is uncertain.
- The profiles and pathway are review-derived and have not undergone external stakeholder validation.

# 13. Priority-reference metadata audit

Four recent or ahead-of-print records were checked against PubMed to confirm their current bibliographic status. Table S4 reports the verified metadata and the relationship between the two Thompson reports.

Table S4. PubMed metadata audit for priority recent references.

| **Current reference** | **Verified PubMed metadata** | **Verification status** |
| --- | --- | --- |
| Ref. 12: Hou et al. | PMID 41573673; Innov Aging. 2026;10(1):igaf136; DOI 10.1093/geroni/igaf136; online December 2025/eCollection 2026 | Year and article number confirmed |
| Ref. 57: Hernandez-Lopez et al. (HELPeN) | PMID 41517343; J Clin Med. 2025 Dec 23;15(1):93; DOI 10.3390/jcm15010093 | Year, volume, article number, and DOI confirmed |
| Ref. 59: Thompson et al., J Clin Nurs | PMID 42261738; DOI 10.1111/jocn.70388; online June 2026 | Ahead-of-print status and DOI confirmed |
| Ref. 60: Thompson et al., J Res Nurs | PMID 41585315; DOI 10.1177/17449871251410561; online January 2026 | Article identifier and DOI confirmed; same eight-nurse parent sample as the J Clin Nurs report |
